# Hotspots and trends of microglia in Alzheimer's disease: a bibliometric analysis during 2000–2022

**DOI:** 10.1186/s40001-023-01602-9

**Published:** 2024-01-24

**Authors:** Lijie Zhang, Qiuru Yao, Jinjing Hu, Baizhi Qiu, Yupeng Xiao, Qi Zhang, Yuting Zeng, Shuqi Zheng, Youao Zhang, Yantong Wan, Xiaoyan Zheng, Qing Zeng

**Affiliations:** 1 Department of Rehabilitation Medicine, Zhujiang Hospital, Southern Medical University, Guangzhou, China; 2 School of Rehabilitation Sciences, Southern Medical University, Guangzhou, China; 3 School of Nursing, Southern Medical University, Guangzhou, China; 4 The First School of Clinical Medicine, Southern Medical University, Guangzhou, China; 5 College of Anesthesiology, Southern Medical University, Guangzhou, China

**Keywords:** Alzheimer’s disease, Microglia, Bibliometrics, CiteSpace, VOSviewer

## Abstract

**Background:**

Alzheimer's disease is one common type of dementia. Numerous studies have suggested a correlation between Alzheimer's disease and inflammation. Microglia mainly participate in the inflammatory response in the brain. Currently, ample evidence has shown that microglia are closely related to the occurrence and development of Alzheimer's disease.

**Objective:**

We opted for bibliometric analysis to comprehensively summarize the advancements in the study of microglia in Alzheimer's disease, aiming to provide researchers with current trends and future research directions.

**Methods:**

All articles and reviews pertaining to microglia in Alzheimer's disease from 2000 to 2022 were downloaded through Web of Science Core Collection. The results were subjected to bibliometric analysis using VOSviewer 1.6.18 and CiteSpace 6.1 R2.

**Results:**

Overall, 7449 publications were included. The number of publications was increasing yearly. The United States has published the most publications. Harvard Medical School has published the most papers of all institutions. *Journal of Alzheimer’s Disease* and *Journal* o*f Neuroscience* were the journals with the most studies and the most commonly cited, respectively. Mt Heneka is the author with the highest productivity and co-citation. After analysis, the most common keywords are neuroinflammation, amyloid-beta, inflammation, neurodegeneration. Gut microbiota, extracellular vesicle, dysfunction and meta-analysis are the hotspots of research at the present stage and are likely to continue.

**Conclusion:**

NLRP3 inflammasome, TREM2, gut microbiota, mitochondrial dysfunction, exosomes are research hotspots. The relationship between microglia-mediated neuroinflammation and Alzheimer's disease have been the focus of current research and the development trend of future research.

**Supplementary Information:**

The online version contains supplementary material available at 10.1186/s40001-023-01602-9.

## Introduction

The most common kind of dementia, Alzheimer's disease (AD) stands as the predominant cause of dementia, potentially accounting for 60–70% of cases. Projections indicate that the global dementia population is expected to nearly double every two decades, reaching 66 million by 2030 and a staggering 115 million by 2050 [1]. Amyloid plaque deposition is a well-established pathological feature of Alzheimer's disease, wherein microglial cells congregate around and infiltrate amyloid plaques within patients' brains, potentially influencing disease pathogenesis [2, 3].

The strong connection between microglia and AD onset, progression, and possible therapies has been shown in several investigations. For instance, a genome-wide association study on the sporadic/delayed AD population identified multiple gene variants that influence microglia-mediated innate immunity [4]. Similarly, integrated systems biology analysis revealed that the microglia-specific immune network was significantly correlated with delayed AD-induced pathological changes [5].

The central nervous system's resident immune sentinels, known as microglia, are found across numerous brain areas [6]. Following changes in the microenvironment, such as ischemia, infection, or injury, microglia become activated and mediate an inflammatory response, disposing of harmful substances and playing a protective role in neurons. However, their excessive release of inflammatory mediators can cause cytotoxic effects [7]. Controlled production of inflammatory mediators can also inhibit or reduce central inflammatory responses and secrete neurotrophic factors, providing further neuron protection [8].

In vitro studies have shown that microglia can phagocytose and clear amyloid-β (Aβ), but Aβ binding to microglial receptors can also activate microglia to release inflammatory cytokines. This interaction accelerates the development of AD because the continued accumulation of Aβ leads to deleterious microglial activation and overproduction of signaling molecules associated with neuroinflammation [9, 10]. This process damages the blood–brain barrier and exacerbates the progression of AD. Therefore, microglial activation is closely related to the development and progression of AD [11].

As an increasing amount of research literature investigates the relationship between microglia and AD, there is a need to synthesize and analyze this information visually to provide valuable insights for future medical research. Therefore, our study aims to provide a comprehensive and scientific analysis of the relevant microglia literature concerning AD, which will enhance our understanding of the current status and future direction of microglia and AD research.

## Methods

### Data collection

**Fig. 1:**
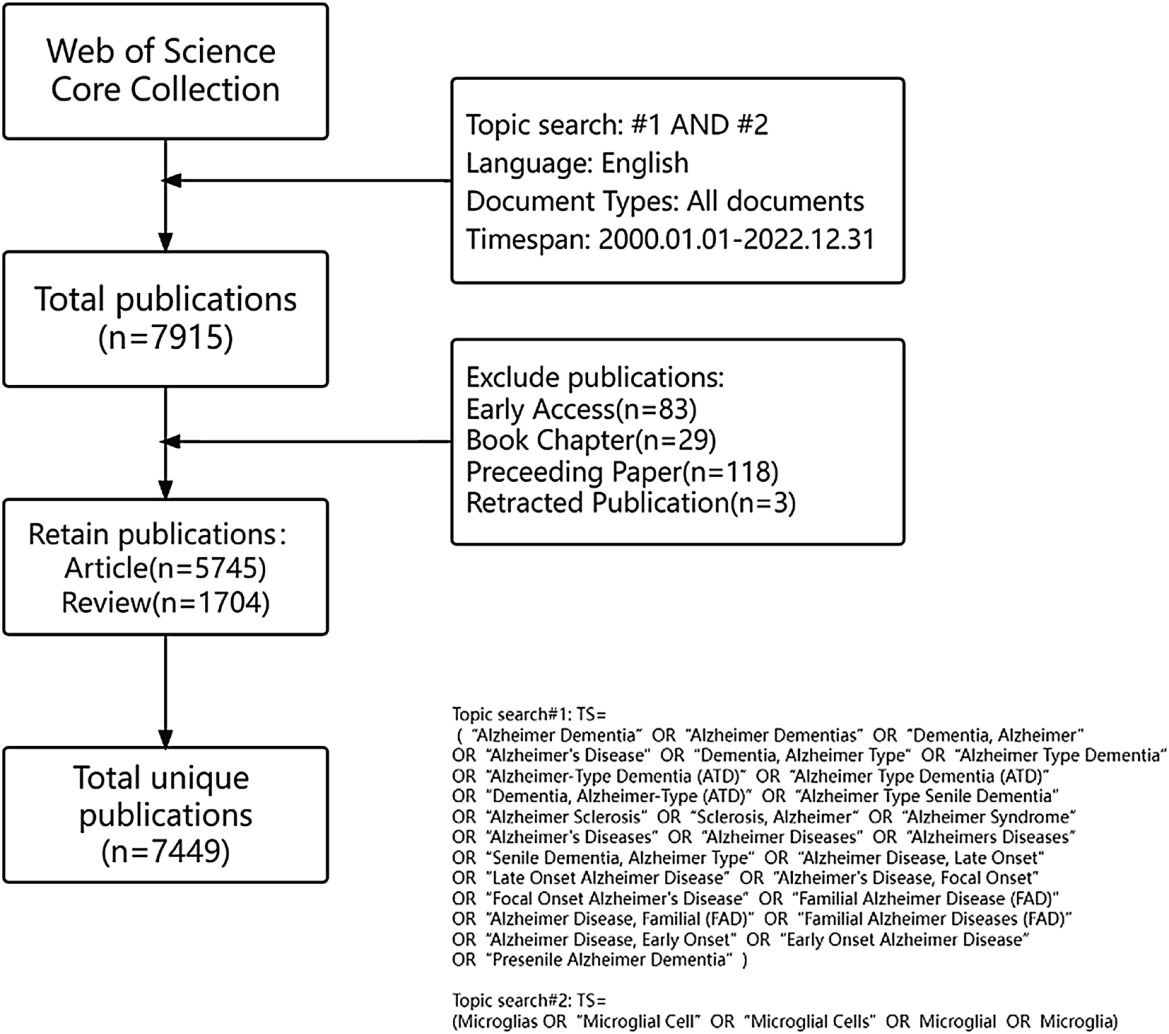
The flowchart of study identification and selection

The Web of Science Core Collection (WoSCC) served as the primary source database for data retrieval. Being one of the largest and most comprehensive online databases in the world, WoSCC offers a vast collection of scientific studies and analyses having high authority and reference significance [12]. In our study, the retrieval formula is set to: TS = (“Alzheimer Dementia” OR “Alzheimer Dementias” OR “Dementia, Alzheimer” OR “Alzheimer's Disease” OR “Dementia, Alzheimer Type” OR “Alzheimer Type Dementia” OR “Alzheimer-Type Dementia (ATD)” OR “Alzheimer Type Dementia (ATD)” OR “Dementia, Alzheimer-Type (ATD)” OR “Alzheimer Type Senile Dementia” OR “Alzheimer Sclerosis” OR “Sclerosis, Alzheimer” OR “Alzheimer Syndrome” OR “Alzheimer's Diseases” OR “Alzheimer Diseases” OR “Alzheimers Diseases” OR “Senile Dementia, Alzheimer Type” OR “Alzheimer Disease, Late Onset” OR “Late Onset Alzheimer Disease” OR “Alzheimer's Disease, Focal Onset” OR “Focal Onset Alzheimer's Disease” OR “Familial Alzheimer Disease (FAD)” OR “Alzheimer Disease, Familial (FAD)” OR “Familial Alzheimer Diseases (FAD)” OR “Alzheimer Disease, Early Onset” OR “Early Onset Alzheimer Disease” OR “Presenile Alzheimer Dementia”) AND TS = (Microglias OR “Microglial Cell” OR “Microglial Cells” OR Microglial OR Microglia). The search was conducted between January 1, 2000, and December 31, 2022, to identify relevant articles for inclusion. We limited our search to English language articles and selected only "Articles" and "Reviews" as the article types. We excluded articles written in other languages, "Proceeding Paper", "Early Access", "Book Chapters", and "Retracted Publication". A total of 7449 articles were retrieved, composed of 5754 articles and 1704 reviews. To avoid potential bias resulting from subsequent database updates, all searches and downloads were completed on a single day, January 21st, 2023. The retrieval process is represented in Fig. 1. All articles were exported in TXT format.

## Data analysis

After verifying the exported data, two authors (LZ and YZ) consolidated redundant elements and rectified spelling inaccuracies. The refined dataset was subsequently imported into VOSviewer 1.6.18 and CiteSpace 6.1 R2 for conducting bibliometric analysis. While VOSviewer and CiteSpace were primarily responsible for conducting the visual analysis of the data, the publication and citation trends of the literature over the years were generated using Microsoft Excel 365.

VOSviewer is a robust bibliometric analysis software that facilitates the extraction and processing of data [13]. It is mainly used for visualizing collaborative networks among countries, institutions, authors, and journals, as well as co-citation of keyword clusters [14]. In addition, CiteSpace is an extensively used bibliometric analysis software that delivers an easy-to-understand comprehension of research hotspots and evolution processes in specific fields, thereby providing insights into future directions for development [15]. CiteSpace is especially useful for analyzing citation bursts and keyword bursts as a means of identifying research hotspots. Additionally, it offers other visual analysis functions, such as clustering for publication data, drawing keyword timeline graphs, and more, that help researchers gain insight into a discipline's past and present life [16].

In our study, we utilized VOSviewer to analyze country/region distribution, institution distribution, author collaboration and distribution, as well as keyword distribution and collaboration. Simultaneously, we employed CiteSpace to analyze the dual-map overlay of journals, reference collaboration and distribution, literature bursts, and keyword bursts.

## Results

### Annual publications and citation trends

**Fig. 2:**
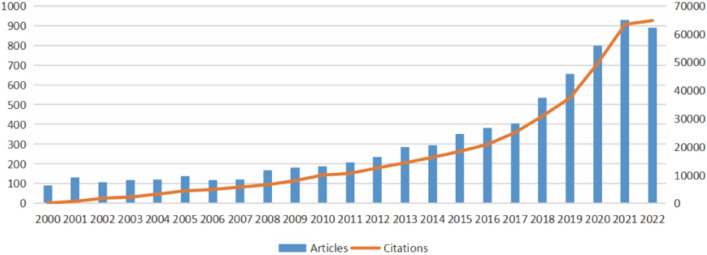
Annual publications and citation trends on research of microglia in Alzheimer's disease

To a certain extent, the number of published documents can indicate the research degree and development overview of the research field. Figure 2 illustrates the number of literatures published from 2000 to 2022 and their corresponding citation trends. It can be observed that from 2000 to 2014, the number of literatures on Alzheimer’s disease steadily increased. However, from 2014 to 2022, the number of publications increased rapidly, with the greatest surge occurring between 2019 and 2020. Obviously, the citation frequency is increasing year by year. Overall, the research of microglia in Alzheimer's disease has demonstrated a prominent trend of rapid progress.

### Distributions of countries/regions

**Fig. 3:**
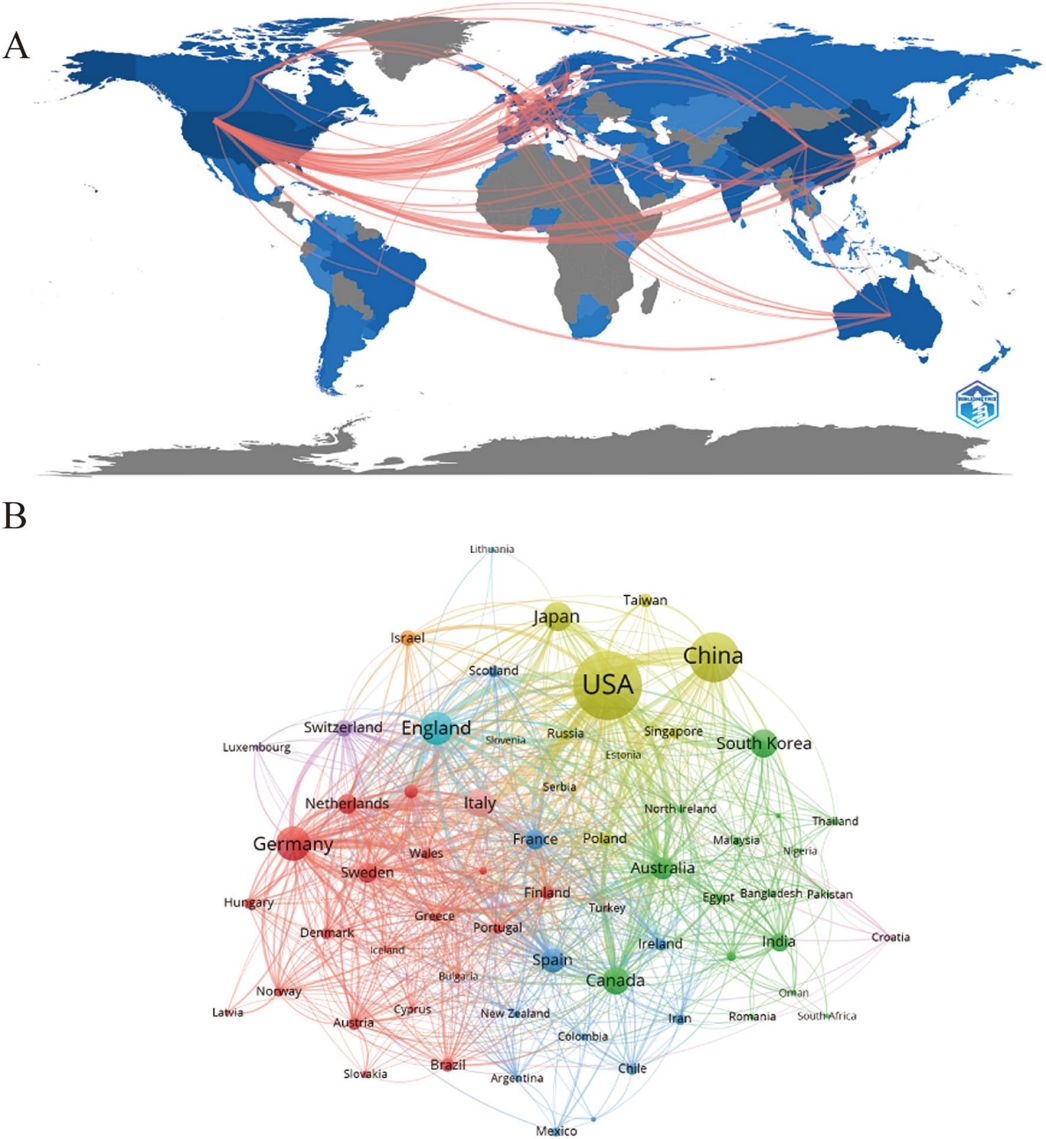
**A** Countries/regions collaboration map. **B** Distributions of countries/regions and collaboration network

At present, 96 countries or regions are engaged in researching microglia in Alzheimer’s disease, with most of the activity concentrated in the northern hemisphere. Furthermore, the links between these countries and regions are primarily located in the northern hemisphere as well. In contrast, Australia, located in the southern hemisphere, demonstrates relatively high activity in this field, maintaining higher frequencies of communication with other countries (Fig. 3A). As shown in Table 1, the top ten countries or regions with the highest number of papers published in this field are led by the United States (2746, 36.86%), with China (1430, 19.19%) following closely behind. The fact that the United States and China, the top two countries in terms of publications, together account for half of the total indicates their significant influence in this field. Following them are Germany (625, 8.93%), the United Kingdom (609, 8.18%), and Japan (460, 6.18%), all of which hold important positions in this area of research. In terms of the overall connection strength, the top five are the United States (1641), the United Kingdom (865), Germany (828), China (506) and Spain (415). We also use VOSviewer to visually analyze countries or regions (Fig. 3B). The degree of cooperation between countries and regions can be classified into ten groups based on their level of collaboration. As evident from the analysis, the level of collaboration between countries and regions is relatively high.

**Table 1:** Top 10 most publication countries/regions related to microglia in Alzheimer's disease

Rank	Country/region	Total link strength	Count (%)
1	USA	1641	2746 (36.86%)
2	China	506	1430 (19.19%)
3	Germany	828	625 (8.39%)
4	England	865	609 (8.18%)
5	Japan	294	460 (6.18%)
6	Italy	355	433 (5.81%)
7	South Korea	168	406 (5.45%)
8	Canada	381	402 (5.39%)
9	Spain	415	312 (4.19%)
10	Australia	319	248 (3.33%)

### Analysis of affiliations

A total of 4,701 institutions have published 7,449 studies on microglia in Alzheimer's disease. Table 2 displays the top 10 institutions based on the number of articles published. There are four institutions that have published more than 100 articles. The most prolific institutions are Harvard Med Sch and Washington Univ (both 130,1.75%), followed by Univ British Columbia(117,1.57%), Univ Calif Irvine(106,1.42%) and Shanghai Jiao Tong Univ(99,1.33%). The University of Washington has the strongest overall correlation strength in this field, followed by Harvard Med Sch. A majority of the top 10 institutions are situated in the United States, underscoring the significant role that American institutions play in maintaining the nation’s prominent position in this field.

**Table 2:** Top 10 most publication institutions related to microglia in Alzheimer's disease

Rank	Institutions	Total link strength	Count (%)
1	Harvard Med Sch	215	130(1.75%)
1	Washington Univ	242	130(1.75%)
3	Univ British Columbia	66	117(1.57%)
4	Univ Calif Irvine	83	106(1.42%)
5	Shanghai Jiao Tong Univ	67	99(1.33%)
6	Case Western Reserve Univ	95	96(1.29%)
7	UCL	158	94(1.26%)
8	Univ Bonn	105	85(1.14%)
8	Univ S Florida	39	85(1.14%)
10	Harvard Univ	105	82(1.10%)

**Fig. 4:**
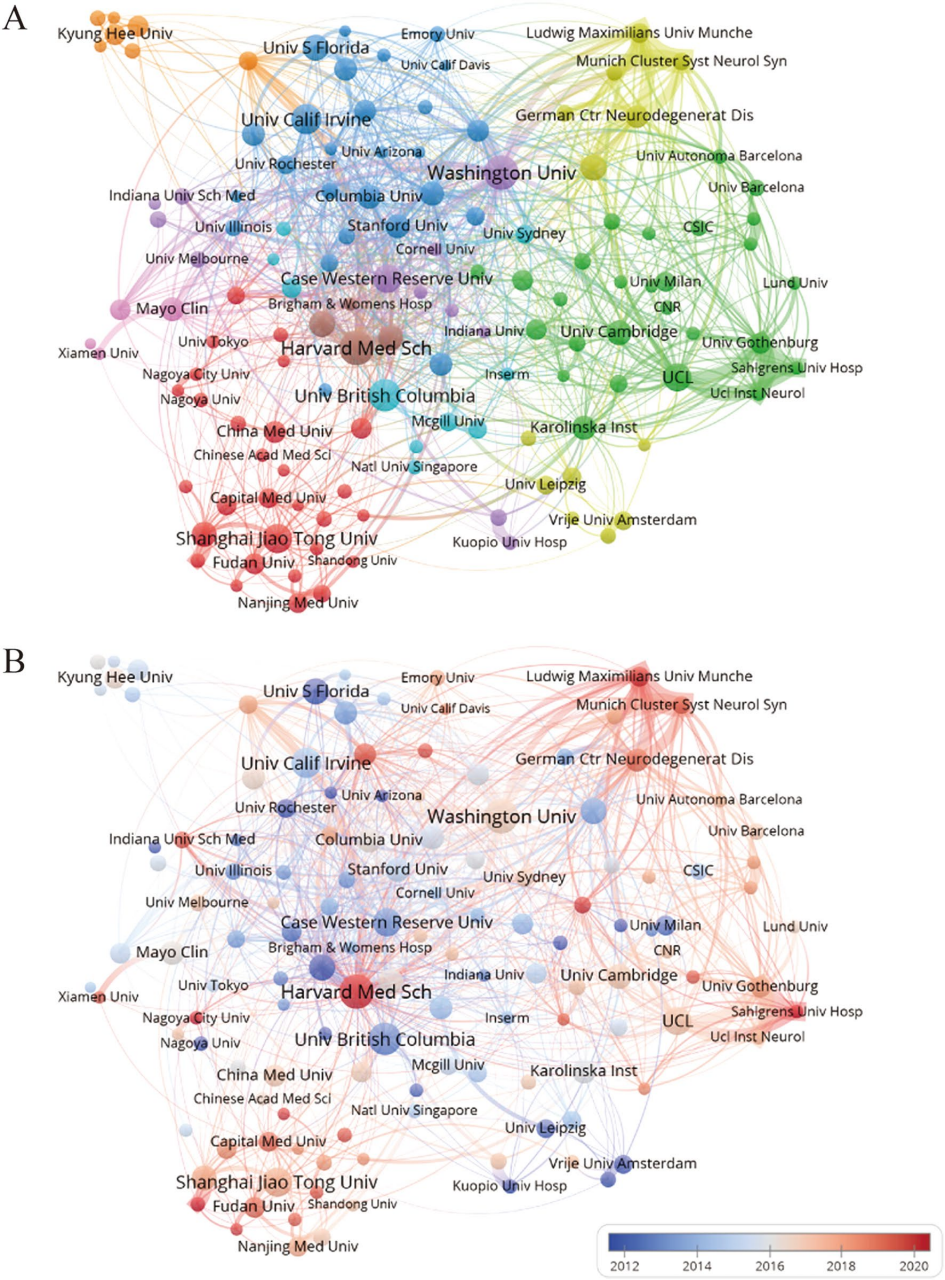
**A** The clustering analysis of the institutions. **B** Network-view map of institutional collaboration

Figure 4A displays the clustering analysis of institutions, while the collaboration network was analyzed visually using VOSviewer. It is evident that these institutions exhibit consistent and close collaboration with each other. Harvard Med Sch, Washington Univ and other institutions have gradually formed a self-centered cooperation network. In Fig. 4B, institutions such as Univ S Florida, Univ Rochester, Brigham & Womens Hosp are mainly blue, and they carried out research in this field earlier. The Chinese institutions, including Shanghai Jiao Tong University, Fudan University, and Capital Medical University, are primarily indicated by light red and red colors, illustrating China’s comparatively recent entry into the field or the production of a significant volume of articles in recent years. Harvard Med Sch has the reddest color and the largest circle nodes, indicating that these institutions may be emerging research organizations in this field, and are likely to dominate future research and become an important force driving the development of the field.

### Contributions of journals

We found a total of 7,449 articles on microglia in Alzheimer's disease published in 203 journals. Table 3 shows that the *Journal of Alzheimer’s disease* had the highest number of published papers (337, 4.52%), followed by the *Journal of Neuroinflammation* (277, 3.72%), and *Neurobiology of Aging* (200, 2.68%). Amid the top 10 journals with the highest number of published papers, five belong to Q1, and five are from Q2, with the *Journal of Neuroinflammation* boasting the highest impact factor (IF) among them, at 9.587. In the top 10 most cited journals, Q1 accounted for 6 and Q2 accounted for 4, indicating that these journals have significant influence and high praise in this field. We can know that there are certain research achievements in this field, and the research level is relatively high as a whole, and there is also room for in-depth research. The impact of a journal largely hinges on the number of citations it receives, as the number of citations reflects the extent to which its articles are being referenced and utilized by scholars and researchers in the field. Among the top 10 cited journals, *Journal of Neuroscience* was cited the most times (21,990), indicating that the journal has an important impact on the study of microglia in Alzheimer's disease, followed by P Natl Acad Sci (14,272) and Usa J Biol Chem (14,002).

**Table 3:** Top 10 journals and co-cited journals related to microglia in Alzheimer's disease

Rank	Journal	Count (%)	IF (2022)	JCR quantile	Co-Cited-Journal	Citation	IF (2022)	JCR quatile
1	Journal of Alzheimer’s Disease	337 (4.52%)	4.160	Q2	J Neurosci	21,990	6.709	Q1
2	Journal of Neuroinflammation	277 (3.72%)	9.587	Q1	P Natl Acad Sci Usa	14,272	12.779	Q1
3	Neurobiology of Aging	200 (2.68%)	5.133	Q2	J Biol Chem	14,002	5.486	Q2
4	International Journal of Molecular Sciences	171 (2.29%)	6.208	Q1	Neurobiol Aging	13,391	5.133	Q2
5	Journal of Neuroscience	169 (2.27%)	6.709	Q1	Nature	12,040	69.504	Q1
6	Frontiers in Aging Neuroscience	159 (2.13%)	5.702	Q1	Neuron	10,123	18.688	Q1
7	Plos One	123 (1.65%)	3.752	Q2	J Neurochem	9780	5.546	Q2
8	Neurobiology of Disease	111 (1.49%)	7.046	Q1	Science	9732	63.714	Q1
9	Journal of Neurochemistry	107 (1.43%)	5.546	Q2	J Alzheimers Dis	8864	4.160	Q2
10	Scientific Reports	104 (1.39%)	4.996	Q2	Glia	8293	8.073	Q1

**Fig. 5:**
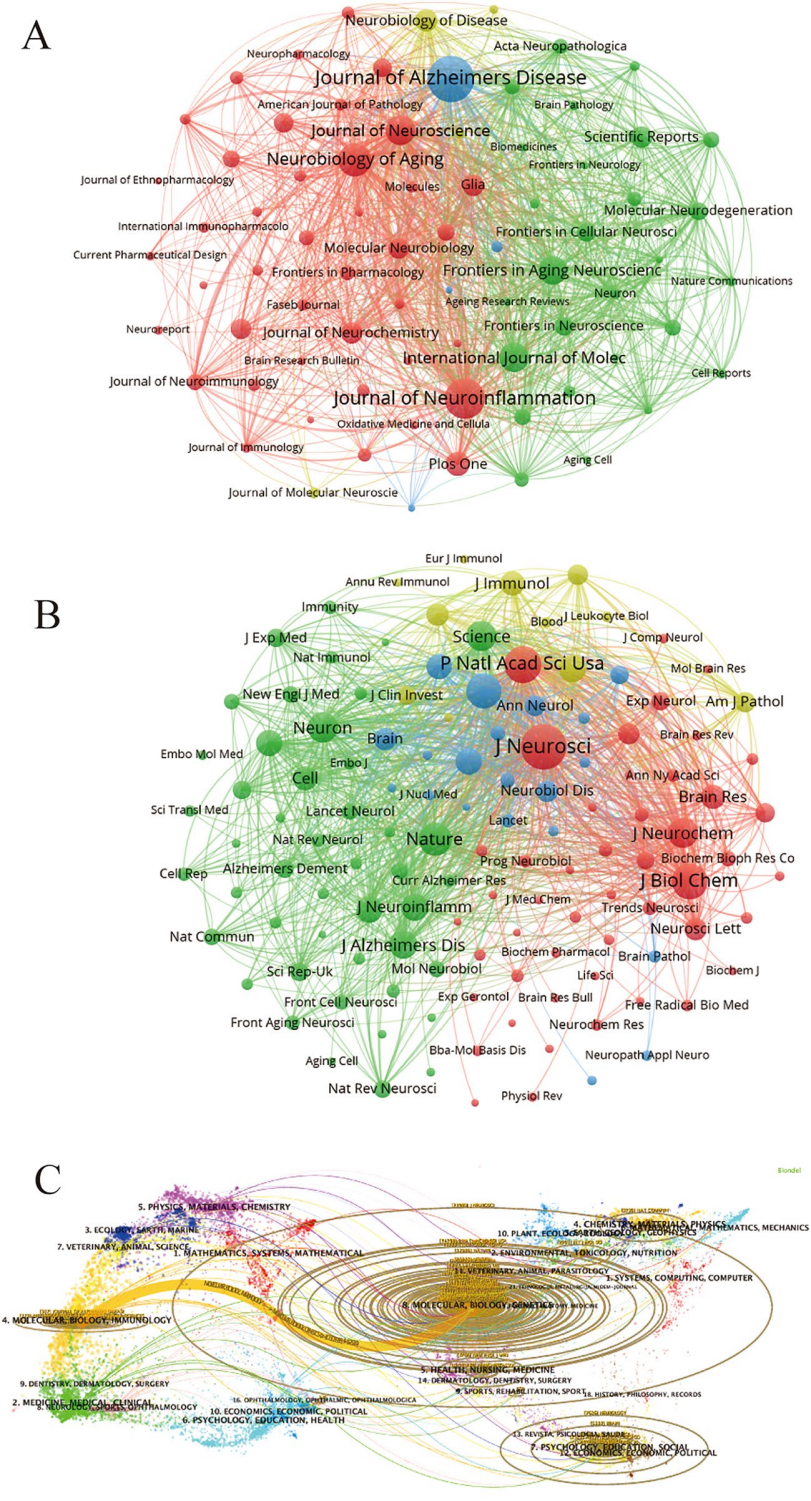
**A** Journals clustering analysis of microglia in Alzheimer's disease. **B** Co-cited journals clustering analysis of microglia in Alzheimer's disease. **C **The dual-map overlay of journal publishing research

We can see the clustering analysis of journals and co-cited journals in Fig. 5A and B, and visually analyze the collaboration network between them with VOSviewer software, which can visually see more detailed cooperation. Periodicals are divided into four clusters according to the frequency of co-citation. Articles in the same journal may have similar research directions or internal logic. We can know that the *Journal of Neuroscience*, *Neurobiology of Aging*, *Journal of Neurochemistry* all have higher co-citation and greater impact. In the dual-map overlay of the journal-published research (Fig. 5C), we observed a prominent cited pathway denoted by yellow. The colored path between the left and right sides depicts the citation relationship between various fields, revealing that studies published in journals focused on molecular, biological, and genetic research primarily cite research published in journals specializing in molecular, biological, and immunological research. This indicates the interdependence and interconnectedness of various fields that contribute to our understanding of Alzheimer’s disease.

## Authors and co-cited authors

Indeed, a total of 29,693 authors are associated with publications related to microglia in Alzheimer's disease. Table 4 provides insight into the top 10 authors based on the number of publications and citation frequency, highlighting the leaders and representative scholars in this field and their contributions to advancing our understanding of Alzheimer’s disease. Heneka, Mt had the highest number of published papers related to microglia in Alzheimer’s disease, with 55 publications, accounting for 0.74% of all publications, followed by Holtzman, David M.(46,0.62%) and Landreth, Ge(45,0.60%). Tan, Lan and Colonna, Marco had higher total link strength, indicating that these authors had many collaborations with other researchers. Among the top 10 co-cited authors, Heneka, Mt(2392) and Mcgeer, Pl(2033) have been cited more than 2,000 times, seven were cited more than 1000 times, suggesting they play a pivotal role in the field.

**Table 4:** Top 10 authors and co-cited authors related to microglia in Alzheimer's disease

Rank	Author	Count (%)	Total link strength	Co-cited author	Citation	Total link strength
1	Heneka, Mt	55 (0.74%)	41	Heneka, Mt	2392	24,503
2	Holtzman, David M	46 (0.62%)	59	Mcgeer, Pl	2033	25,003
3	Landreth, Ge	45 (0.60%)	39	Selkoe, Dj	1386	12,573
4	Klegeris, Andis	42 (0.56%)	17	Streit, Wj	1277	16,540
5	Zetterberg, Henrik	39 (0.52%)	41	Akiyama, H	1187	14,022
6	Colonna, Marco	37 (0.49%)	62	Wyss-Coray, T	1111	14,160
7	Blennow, Kaj	33 (0.44%)	38	Braak, H	1044	9117
8	Tan, Lan	33 (0.44%)	65	Rogers, J	819	12,405
9	Saido, Takaomi C	32 (0.42%)	54	Hardy, J	815	8109
10	Lue, Lih-Fen	30 (0.40%)	39	Hickman, Se	786	10,545

**Fig. 6:**
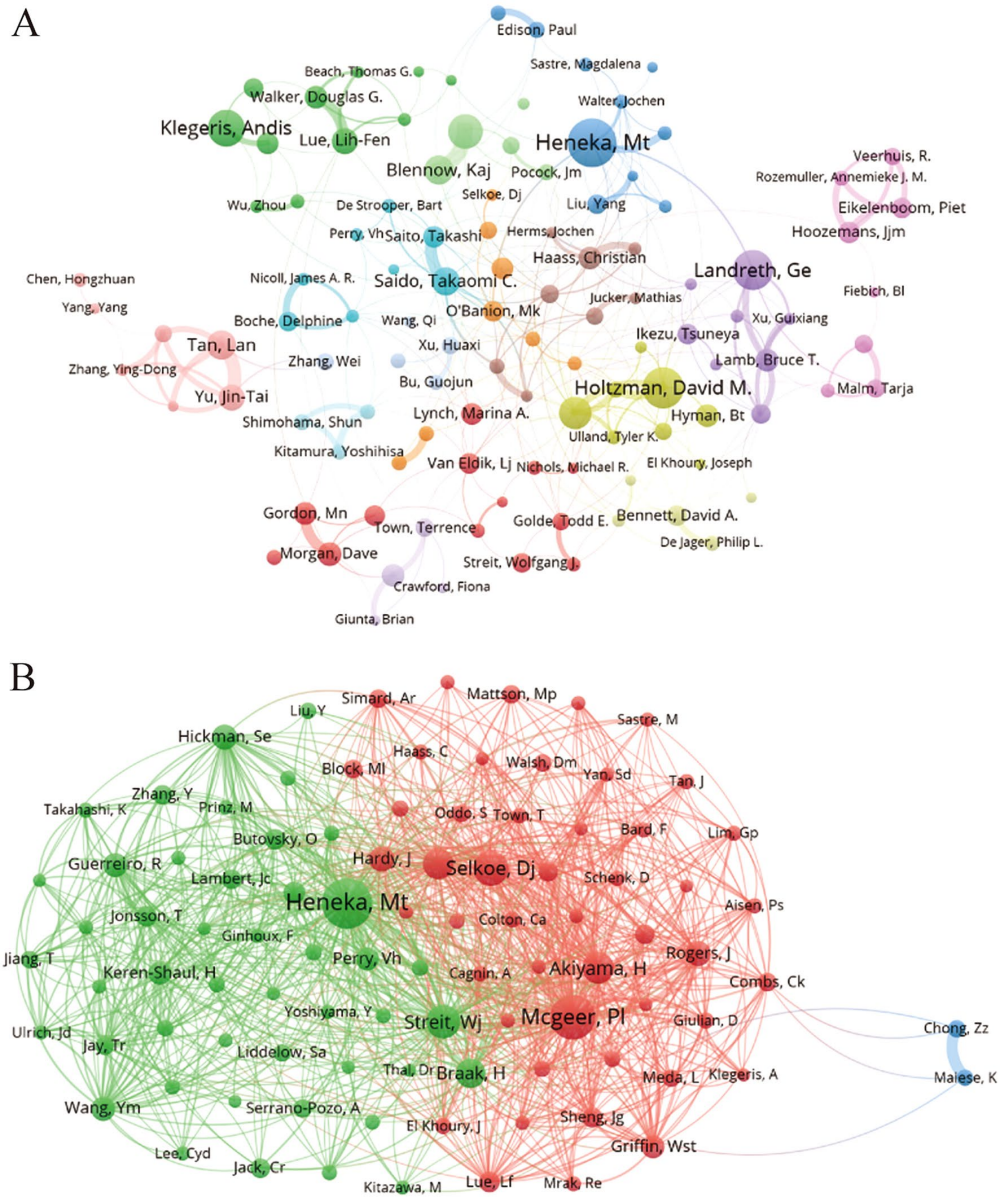
**A** Co-author related to microglia in Alzheimer's disease. **B** Network of co-cited authors. The size of the node depends on the author's co-citation

A collaborative network of authors in the literature on microglia in Alzheimer's disease was visualized with VOSviewer, with different colors representing different clusters. The co-authors formed 14 clusters (Fig. 6A). The brown group (Haass Christian, Herms Jochen, Jucker Mathias), dark blue group (Heneka Mt, Liu Yang, Walter Jochen) and yellow group (Holtzman David M, Ulland Tyler K, Hyman Bt) have more extensive contact with the outside world. Figure 6B displays the collaboration network of co-cited authors, which is partitioned into three distinct clusters. Only the blue group (Chong Zz, Maiese K) had sparse connect with the other two groups. Heneka Mt has the most significant amount of co-citations, trailed by Mcgeer PI and Selkoe Dj, who are representatives and core research forces in the field and enjoy a high academic reputation.

### 3.6 References and co-cited references

Additional file 1 displays the 15 most frequently cited articles from the pool of 7449 retrieved papers. "Inflammation and Alzheimer’s disease" is the most cited article (3439 citations). The objective of this paper is to gain further insight into the immunomodulatory and inflammatory mechanisms in Alzheimer’s disease and to aid in the advancement of novel anti-inflammatory methods, with the aim of decelerating the disease progression. The paper endeavors to furnish proof for the pathological significance of inflammation in Alzheimer’s disease and to explore intricate interactions between agents of inflammation to develop fresh ideas and therapeutic approaches for alleviating inflammation in Alzheimer’s disease [17]. The second most cited article is "The amyloid hypothesis of Alzheimer’s disease at 25 years" (Selkoe et al. 2016) with 3028 citations. This article reviews new developments in the amyloid-beta hypothesis for Alzheimer's disease, summarizes more than 30 pieces of evidence to support this hypothesis, and discusses related concepts and concerns [18]. The authors of the article, “Neuroinflammation in Alzheimer’s Disease”, which has the third-highest number of citations at 2,985, offer a synopsis of the role of neuroinflammation in Alzheimer’s disease. The article discusses the relevant cell types and mediators involved, as well as techniques applied to visualize neuroinflammation, and the clinical implications, along with prospective therapeutic approaches [19]. “Delivery of siRNA to the mouse brain by systemic injection of targeted exosomes”, the fourth most cited article with 2,783 citations, validates the therapeutic potential of utilizing exosome-mediated RNA interference for Alzheimer’s disease. The study demonstrates the effectiveness of this approach by successfully knocking down 60% and 62% of BACE1 mRNA and protein, respectively, a significant therapeutic target for Alzheimer’s disease, in wild-type mice via systemic injection of targeted exosomes [20].

**Fig. 7:**
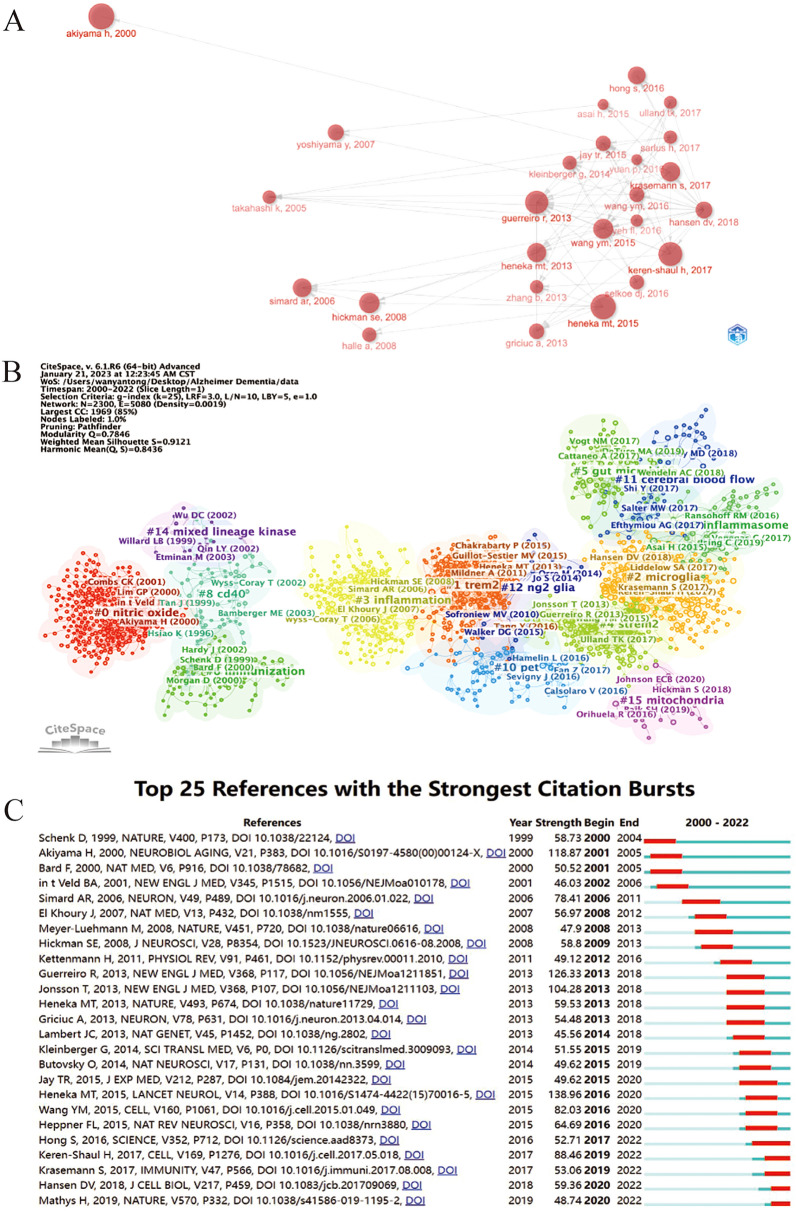
**A** The network diagram of document relationship obtained by VOSviewer. **B** Cluster view of references on microglia in Alzheimer's disease. **C** CiteSpace visualization map of top 25 references with the strongest citation bursts involved in microglia in Alzheimer's disease

By analyzing the relationship between studies and citations of literature, the cited literature was co-cited analysis (Fig. 7A), and the relationship network of the literature was clustered with CiteSpace, resulting in a total of 15 co-reference clusters (Fig. 7B). We found a specific time to publication factor that marked an explosive citation frequency in the literature. The earliest outbreak articles were in 2000, 2013, 2016 and 2017 with a higher number of explosive studies and citations from published studies. Articles with a substantial citation frequency contribute considerably to the group of explosive articles, thereby demonstrating the correlation between explosive research and citation frequency. The cluster diagram, as illustrated in Fig. 7B, exhibits a modularity Q value of 0.7846 and a weighted mean silhouette value of 0.9121, which confirm a robust cluster structure and provide strong evidence for the stability and accuracy of the clustering analysis. Cluster #0 was the biggest cluster, and its size signifies the number of times the papers linked to it have been cited. The keyword with the most significant frequency across all clusters is “nitric oxide”, followed by “TREM2” (cluster #1), followed by “microglia” (cluster #2), “inflammation” (cluster #3), and “sTREM2” (cluster #4). Other important clusters are “gut microbes”, “immunization”, “NLRP3 inflammasome”, and “CD40”. Combined with Fig. 7A, from the perspective of time changes, researchers have paid more attention to “gut microbes”, “microglia”, “mitochondria” in recent years, which, in a sense, may imply a shift.

Figure 7C outlines the top 25 references that have the highest citation bursts concerning microglia in Alzheimer’s disease. This list enables us to track research hotspots and their durations. By analyzing the articles with high explosive intensity, we can better grasp the direction of research development. The article “Neuroinflammation in Alzheimer's disease” published in LANCET NEUROLOGY by Heneka MT et al. in 2015 had the strongest burst (strength = 138.96) and the duration of the outbreak was from 2016 to 2020 [19].

## The analysis of hotspots and frontiers

### Keywords co-occurrence and cluster analysis

**Fig. 8:**
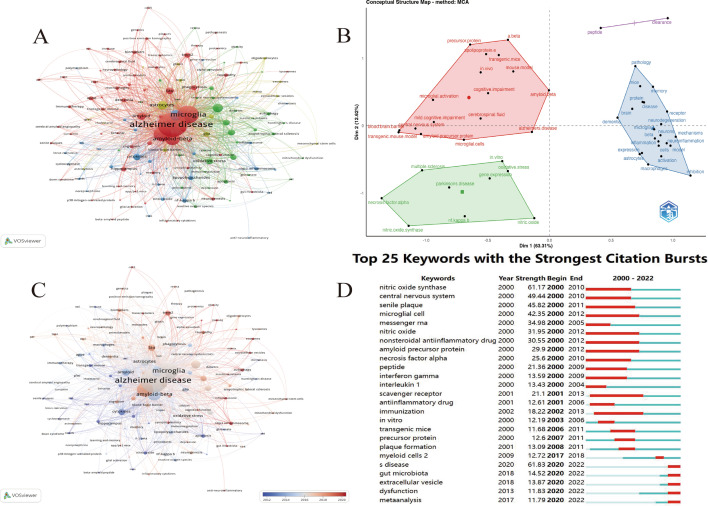
**A** The keyword co-occurrence network relationship clustering diagram. **B** A map of the conceptual structure of the top 50 terms in terms of word frequency. **C **Network-view map of keyword co-occurrence. **D** CiteSpace visualization map of top 25 keywords with the strongest citation bursts

The clustering of keywords is shown in Fig. 8A. The keyword clustering analysis yielded eight clusters, with the primary focus of the red cluster being on “Alzheimer’s disease”, “aging”, and “amyloid”. The green cluster contains primary keywords such as “TREM2”, “tau”, and “dementia”. The orange cluster includes keywords like “inflammation”, “cytokines”, and “nitric oxide”. Other important keywords include “neurodegeneration”, “astrocytes”, “amyloid-beta”. Indeed, the identification of these keywords and their clusters can help in revealing the current research hotspots and frontiers, and can offer a reference for future research in the field. It gives an insight into the most notable and emerging areas of research for Alzheimer’s disease, such as the role of TREM2 and tau in the development of dementia, the involvement of inflammation and cytokines, and the relationship between aging and amyloid, which can help researchers direct their focus towards these key areas of research.

Figure 8B shows the results of the multiple correspondence analysis through the MCA function of the FactoMineR package. X and y coordinates of the figure are dim1 and dim2 obtained from the MCA analysis, and the values in parentheses are the feature accumulation percentages. Therefore, the figure is a combined graph of clustering and MCA analysis of keywords. The visual representation of keyword MCA analysis can qualitatively determine the similarity of different keywords based on the distance between keywords, and the role of clustering here is to make this similarity of keywords more explicit. Obviously, Fig. 8B shows that the top 50 ranked keywords can be divided into 4 categories, and the greater similarity of keywords in the same category may represent a topic, and interpreting the topic requires certain knowledge in the field or consulting experts.

### Keywords timeline viewer

Figure 8C displays the analysis of the mean year of publication for each keyword, with a change from blue to red represents the temporal progression. Among the high-frequency keywords such as “Alzheimer”, “neuroinflammation”, “neurodegeneration”, “microglia”, “astrocytes”, and “Parkinson’s disease”, the mean year of publication for Alzheimer is 2015, and the rest of the keywords were also used at a higher frequency in the field from 2015 to 2017. We can observe that the use of keywords has evolved over time, with “cytokines” and “chemokines” in 2012, “Alzheimer”, “microglia”, and “astrocytes” in 2015, and “neuroinflammation”, “neurodegenerative diseases”, “tau”, and “TREM2” in 2017 and 2018. The keywords have progressed to include “RNA-seq”, “extracellular vesicles”, and “gut microbiota” in the past two years. The aforementioned observation implies that the research orientation in this domain has undergone a transformation over time, contributing to enlightening our perception of the research process and to our ability to apprehend the future research frontier.

#### Burst detection

Keywords are refined and extracted from the article, which is the label of a paper, and keywords with high frequency can reflect the main research directions and hot spots in the field. Table 5 exhibits the 20 most frequent keywords found in literature concerning microglia in Alzheimer’s disease. The top five keywords extracted from Table 5 are “Alzheimer” (4069), “microglia” (2308), “neuroinflammation” (1517), “amyloid-beta” (1070), and “inflammation” (833). The high frequency of these keywords suggests that research related to these topics is currently at the forefront of research in this field. The term “outbreak keyword” is a metric employed to highlight the most active research areas in a specific field. If a keyword is still in its outbreak period in recent years, it indicates that the research related to that keyword may be at the forefront of research and represent the research frontier in that field. As shown in Fig. 8D, the top 25 keywords with the highest outbreak intensity include “gut disease”, “extracellular vesicle”, “dysfunction”, and “meta-analysis”. The fact that these keywords are still in their outbreak period until 2022 implies that research in these areas may represent the current research frontier in this particular field. This suggests that the research direction related to these keywords may still be a hotspot in the field.

**Table 5:** Top 20 keywords related to microglia in Alzheimer's disease

Rank	Keyword	Occurrences	Total link strength	Rank	Keyword	Occurrences	Total link strength
1	Alzheimer	4069	9804	11	Parkinson's disease	291	949
2	Microglia	2308	6576	12	Oxidative stress	246	678
3	Neuroinflammation	1517	4227	13	Neurodegenerative diseases	230	628
4	Amyloid-beta	1070	2998	14	Aging	226	676
5	Inflammation	833	2397	15	Neuroprotection	221	591
6	Neurodegeneration	552	1691	16	Trem2	208	618
7	Astrocytes	538	1794	17	Phagocytosis	207	627
8	Amyloid	369	1188	18	Lipopolysaccharides	148	403
9	Tau	321	1070	19	Hippocampus	146	403
10	Cytokines	305	1072	20	Dementia	140	434

## Discussion

In this study, we evaluated the hotspots and development patterns in the study of microglia in Alzheimer's disease. A total of 7449 articles between 2000 and 2022 from the Web of Science were retrieved. The data were examined using CiteSpace 6.1. R2 Advanced, VOSviewer 1.6.18, and R bibliometrix.

### General information

The yearly amount and trend of literature might provide insight into the study's development and research progress [21]. Figure 2 reveals that from 2000 to 2007, the number of papers fluctuated around 100 per year, indicating that research on microglia was still in its early stages. The substantial rise in publications on microglia in Alzheimer's disease from 2018 to 2022 suggests that this area of research is becoming more and more important. It is important to point out that over half (50.12%) of the total publications occurred in the last five years (2018–2022), demonstrating that microglia in Alzheimer’s disease has been drawing increasing interest from scholars in recent years.

From the distribution of countries/regions, we can observe that the United States has the most published articles as well as the most citations, which indicates its dominant position in this field. Notably, in terms of the number of literatures, the United States and China together account for 56.05% of the total (USA 36.86%, China 19.19%), indicating that these two countries are currently leading in the research on microglia in Alzheimer’s disease. Moreover, six of the top ten institutions in terms of publications are from the United States; eight of the top ten institutions in terms of citation frequency are also from the United States. These findings all suggest that the United States plays a decisive role in the development of this field.

As shown in Table 3, *Journal of Alzheimer’s Disease* published the most studies on microglia in Alzheimer’s disease, and was also ranked ninth among the most cited journal. Four of the top 10 journals with the most publications are also among the top 10 co-cited journals (*Journal of Alzheimer’s Disease*, *Neurobiology of Aging*, *Journal of Neuroscience*, *Journal of Neurochemistry*), demonstrating their greater significance in the area. Furthermore, it is worth mentioning that all the top 10 journals are Q1 or Q2, which indicates that the quality of publications in this area is generally high. Five of the top 10 journals are primarily in the neurosciences, while others have a relationship with biochemistry, molecular biology, and interdisciplinary fields according to co-citation frequency. This is in line with dual-map analysis.

In accordance with Table 4, Heneka, Mt has the most papers (55, 0.74%) and citations (2392 citations), indicating his greatest influence and most outstanding contributions to the field of microglia in AD. Heneka, Mt, a professor of Bonn University, specializes in Neurodegenerative Diseases, Neurodegeneration, and Molecular Biology. In 2013, Heneka, Mt et al.[22] published the paper in Nature, titled “NLRP3 is activated in Alzheimer's disease and contributes to pathology in APP/PS1 mice”, which confirmed the involvement of NLRP3 in the onset of AD. This article was listed in the top 25 references bursts with the citation strength and ranked ninth among the top 10 cited references. In 2015, Heneka, Mt and his team [19] systematically reviewed the involvement of neuroinflammation in the development of AD, which ranked third (2985 times co-citation) and had the strongest citation bursts (138.96). In 2008, a Hickman, Se’s article published by *Journal of Neuroscience* concluded that pro-inflammatory cytokines produced by amyloid-β deposition can impair microglial clearance function as AD progresses [23], which provided ideas for Heneka, Mt to study the effect of NLRP3 deficiency on the phagocytosis of microglia in vivo. The article was listed in the top 25 references bursts with the citation strength.

### Hotspots and frontiers

As they represent the main research subjects of a particular field, keywords and references are crucial components of scholarly literature. Reference clusters and citation bursts can provide insights into newly popular subjects within the discipline [24, 25]. Among the top 10 co-cited references, four are associated with neuroinflammation [17, 19, 22, 26]; one pioneered the identification of a new type of microglia ( DAM) and explored the way it is activated [27]; one reveals that TREM2 variation has a strong link with an increased risk of AD [28], one explores the treatment of AD by injection of targeted exosomes [20]; one emphasizes the critical function of the brain microenvironment in the onset and progression of AD [29]; one is mainly on amyloid protein hypothesis [30]. Finally, one put forward progranulin mutations were identified as the cause of neurodegenerative diseases, based on the increased expression of progranulin in activated microglia [31]. Moreover, according to Fig. 7C, five references are still in burst and worthy of our attention. Two of them concentrated on how microglia affected progression of AD [32, 33], while the other three explored new pathways and novel cell subpopulations [27, 34, 35]. Furthermore, as pictured in Fig. 7B, the earliest and largest cluster is #0 (nitric oxide). It is notable that several topics have garnered continuous attention in recent years, including #5(gut microbiota), #11(cerebral blood flow), #7(NLRP3 inflammasome), #15 (mitochondria) which are all related to the pathologic mechanism of AD.

As presented in Table 5, the keywords with high occurrence frequencies, aside from the search terms “Alzheimer” and “microglia”, include neuroinflammation are “neuroinflammation” (1517), “amyloid-beta” (1070), “inflammation” (833), “neurodegeneration” (552), “astrocytes” (538). High-frequency keywords indicate plenty of popular research directions, including inflammation (neuroinflammation, oxidative stress), immunology (cytokines, phagocytosis, lipopolysaccharides), neuroscience (neurodegeneration, neuroprotection). Based on Fig. 8C, “TREM2”, “exosomes”, “autophagy”, “NLRP3 inflammasome”, “gut microbiota”, “tauopathy”, “RNA-seq”, “synaptic plasticity” are hot words in the past two years, representing the frontiers and future development direction. These keywords are mainly related to the pathogenesis and treatment of AD.

#### The effect of microglia-mediated neuroinflammation on AD progression

On the basis of keyword occurrence, chronology analysis, reference clustering, as well as the top 10 co-cited references, it can be concluded that neuroinflammation continues to be a hotspot and frontier in this field. The earliest popular inflammation-related research was on nitric oxide (Fig. 7B). An article [36]published by Comb CK showed that Aβ stimulates microglia to produce tumor necrosis factor-alpha (TNF α), inducing a large number of inducible nitric oxide synthase and causing neuritic dystrophy of neurons that were mainly dependent on neurotrophins [37, 38]. The findings of reference clustering reveal that neuroinflammation was most strongly associated with the NLRP3 inflammasome. The NLRP3 inflammasome, as molecular sensors in microglia, is activated by signals that have the hallmarks of AD(such as Aβ) [39]. The general consensus is that two signals are necessary for NLRP3 inflammasome activation [40, 41]. The nuclear factor kappa B (NF-κB)-dependent transcription of NLRP3 and pro-IL-1 is the priming signal, and it is initiated by the interaction of the TLR4 ligand LPS to its receptor. The NLRP3 complex is assembled and activated by the second signal (the activation signal), which is triggered by extracellular ATP, certain bacterial toxins, crystalline and particulate materials. Moreover, there is evidence that Aβ activates NLRP3 inflammasomes through the Syk–AMPK pathway [42]. Microglia cells produce interleukin (IL)-1 and IL-18 through caspase-1, which leads to neuroinflammation and neuronal death when the NLRP3 inflammasome is activated [43, 44].

The connection between TREM2, gut microbiota, mitochondrial dysfunction, exosomes, and inflammation has drawn more attention in recent years. Microglia prominently express triggering receptor expressed on myeloid cells-2 (TREM2) [45]. TREM2 inhibits inflammatory responses by inhibiting microglia-mediated cytokine production and secretion [46]. However, there is evidence that CD45 ( hi) Ly6C ( +) macrophages were almost eliminated in AD mice lacking TREM2, thereby reducing inflammation [47]. Jay, TR [48] and Karanfilian, L [49] provided an explanation: TREM2 reduces pro-inflammatory cytokine gene expression in microglia in the early stages of AD. In the latter phases, however, when microglia are persistently activated and secrete more pro-inflammatory cytokines, inflammation decreases in TREM2-deficient mice. The anti-inflammatory mechanism induced by TREM2 may be related to the PI3K–FoxO3a axis [50]. Recent research has demonstrated that TREM2 can regulate lipid metabolism, thereby promoting inflammation and aggravating AD progression [51]. Furthermore, Galectin-3, a new endogenous TREM2 ligand, was shown for the first time by Boza-Serrano A [52] to enhance inflammation and aggravate disease progression by raising microglia activation. Another popular topic in recent years is soluble TREM2 (sTREM2), a proteolytic product. It has been demonstrated that STREM2 mediates inflammatory responses and preserves microglial cell viability [53]. By activating the NF-κB signaling pathway, STREM2 boosts microglial cell survival and increases the production of inflammatory cytokines [54, 55]. Wild-type sTREM2 prevents Aβ from folding into an aggregate form and refolds the aggregate into a soluble form, thereby inhibiting the neurotoxicity of Aβ [56]. Whether sTREM2 has a protective effect on Alzheimer 's disease is still controversial.

The significant driving component to the development of AD is microglia-driven neuroinflammation [57], whose mechanism is connected to microglial mitochondrial dysfunction [58]. The endo-lysosomal membrane's TLR9 may bind to oxidized mitochondrial DNA, activating the NLRP3 inflammasome and IFN-related pathways [59]. Moreover, microglia with mitochondrial DNA damage generate a lot of ROS, which activates the NF-κB signaling pathway and results in a plenty of pro-inflammatory cytokines [60]. Additionally, the decrease of mitophagy in microglia will increase the release of harmful contents and amplify the inflammatory response [61, 62]. The regulation of autophagy is related to mTOR signaling [63]. In light of these mechanisms, a viable treatment target for AD currently is mitochondrial dysfunction [64, 65].

In recent years, study on the links between gut microbiota and neuroinflammation has drawn considerable interest. Chun Chen et al. showed that increased bacteroides in the gut microbiota of AD patients mediates pro-inflammatory poly-unsaturated fatty acid metabolism and regulates microglial activation in the brain [66]. Similarly, after intestinal barrier injury, pro-inflammatory factors produced by gut microbiota enter the body and activate microglia-induced neuroinflammation through the LPS/TLR4/NF-κB/NLRP3 inflammasome pathway [67–70]. Meanwhile, LPS stimulates the vagus nerve, which is an essential part of the gut–brain axis [71], and activates microglia in the brain, inducing neuroinflammation [72]. Another recent study observed that the gut microbiota of AD patients worsened AD pathologies in 3 × Tg mice, which correlated to the activation of the C/EBPβ/asparagine endopeptidase pathway [66].

One of the latest research hotspots is the correlation between exosomes and neuroinflammation. However, the effect of exosomes on AD progression remains controversial. On the one hand, microglial exosomes can carry tau [66], Aβ [67, 68], pro-inflammatory signals to neighboring neurons, triggering exosome-mediated propagation of toxic species. On the other hand, exosomes from other sources can internalize Aβ and transport it to microglia or lysosomes for clearance [69, 70]. Exosomes from M2 microglia are also documented to reduce neuronal damage and mitochondrial dysfunction in AD through the PINK1/Parkin pathway, thereby exerting neuroprotective effects [71].

#### Microglia-mediated synaptic loss causes excitatory/inhibitory synaptic imbalance in AD

Synaptic plasticity and tauopathy have received more attention lately (Fig. 8C). Alzheimer's disease is the most prominent secondary form of tauopathy [72]. It is worth noting that synaptic loss and microglial activation take place in a mouse model of P301S tauopathy prior to the development of tangles [73]. Loss of synaptic connections is directly associated with cognitive impairment, one of Alzheimer's disease's primary symptoms [74]. Synaptic loss caused by aberrant microglial activation in a diseased situation is mostly explained by the classical complement cascade [75, 76]. Pathological Aβ or tau may cause an initial protein of the classical complement cascade, C1q, to be upregulated in microglia [77, 78]. When C3 is deposited on synapses as a result of C1q activation, it binds to CR3 on microglia and causes them to phagocytose synapses [79, 80]. In addition, evidence suggests that ApoE4 has the potential to exacerbate this effect by increasing the accumulation of C1q, leading to excessive activation of microglia and increased synaptic loss[81], which is related to transport lipid and mediate lipid toxicity [27, 82]. C1q and C3, as "eat me" signals, promote microglial phagocytosis. In contrast, the "don't eat me" signal CD47 counters this effect [83, 84]. Correspondingly, CD47-deficient mice were found to undergo excessive pruning of microglia, leading to a continuous decrease in the number of synapses [84]. Furthermore, studies have shown that TREM2-deficient mice have increased dendritic spine density and enhanced electrophysiological activity, which interferes with the role of microglia in synaptic modification through phagocytosis [85, 86]. TREM2 may sense damaged synapses in AD through the phosphatidylserine (PS), which represents a neuronal “eat-me” signal [87]. However, the specific mechanism remains to be further studied. More precisely, the complement–microglia axis and Aβ-induced inhibitory synaptic loss rather than excitatory synapses, leading to the imbalance of excitatory/inhibitory synapses and cognitive decline [88–90]. Remarkably, microglia expressing GABAB receptor selectively mediate inhibitory synaptic remodeling [91], contributing to excitatory/inhibitory imbalance in AD [92]. Furthermore, the exposure of phosphatidylserine leads to the loss of inhibitory postsynaptic specificity, resulting in abnormal excitability and seizures [93]. In addition, the moderate amount of Aβ in the AD brain cause abnormal glutamate receptor activation, which eventually induces hyperexcitability and degeneration of glutamatergic and cholinergic neurons [94, 95]. On the one hand, brain regions with abnormal neural network are strongly consistent with fragile regions in AD [96–98]. On the other hand, regulating excitatory/inhibitory imbalance can effectively improve brain rhythm and cognitive function [99, 100]. These two reasons provide evidence that excitatory/inhibitory imbalance promotes the progression of AD. Importantly, excitatory/inhibitory imbalance disrupt the mechanism of brain communication, resulting in cognitive impairment [101].

## Limitation

This study analyzed the research trends in a specific area by examining articles from the Web of Science core collection of SCIE-indexed English core journals. Although the results provide valuable insights, this study does have several limitations. Firstly, the study may be missing articles published in other databases or languages, making it important for future studies to extend their coverage by utilizing databases such as PubMed or Scopus. Moreover, keyword and reference analysis may not provide enough information to reveal deeper research motivations and specific research processes. Additionally, as older articles tend to have higher citation rates, it may be difficult for newer high-quality literature to rank in the top 10. Lastly, it is important to note that bibliometric analysis is better suited to extracting macro-level trends rather than medium- and micro-level analyses.

## Conclusion

A total of 7449 publications concerning the role of microglia in Alzheimer's disease have been published worldwide in the last two decades, demonstrating a general trend of annual increase and suggesting a high level of interest in related research fields. In terms of publications, United States and China are currently leading in the research. Harvard Medical School has published the most papers of all institutions. Heneka MT is the most influential author with the most articles and cited times. Specifically, the research has mainly focused on neuroinflammation and excitatory/inhibitory synaptic imbalance. Neuroinflammation mediated by microglia might be one of the primary causal factors contributing to the occurrence and development of AD. Although the significance of neuroinflammation in the pathogenesis and treatment of AD remains controversial, researchers have given substantial attention to exploring the link between TREM2, gut microbiota, mitochondrial dysfunction, exosomes, and inflammation. Consequently, NLRP3 inflammasome, TREM2, mitochondrial dysfunction, and gut microbiota have become promising therapeutic targets for AD. Additionally, utilizing exosomes to deliver genes or drugs to target sites could represent a promising therapeutic direction. Current studies center on the effects of microglia-mediated synaptic loss resulting from excitatory/inhibitory synaptic imbalance. It is essential that future studies continue to explore the role of microglia in the development of AD; this not only helps to elucidate the cellular and molecular mechanisms of neurodegeneration, but also may provide new ideas and targets for the treatment and early intervention of neurodegenerative diseases.

### Supplementary Information

**Additional file 1: Table S1.** Top 15 co-cited references related to microglia in Alzheimer's disease.

## Data Availability

The original contributions presented in this study are included in the article/Supplementary material. Further inquiries can be directed to the corresponding authors.
