# Supplementary material for: Hotspots and trends of microglia in Alzheimer's disease: a bibliometric analysis during 2000–2022
Source: Eur J Med Res. 2024 Jan 24;29:75. doi: 10.1186/s40001-023-01602-9 (PMC10807212; doi:10.1186/s40001-023-01602-9)
Supplement: Supplementary file 1 — Additional file 1: Table S1. Top 15 co-cited references related to microglia in Alzheimer's disease. [file 40001_2023_1602_MOESM1_ESM.docx]

Additional file 1: Table S1: Top 15 co-cited references related to microglia in Alzheimer's disease.

| **Rank** | **Author** | **Title** | **Source** | **Citation** | **Year** | **DOI** |
| --- | --- | --- | --- | --- | --- | --- |
| 1 | Akiyama,H,et al. | Inflammation and Alzheimer's disease | NEUROBIOLOGY OF AGING | 3439 | 2000 | 10.1016/S0197-4580(00)00124-X |
| 2 | Selkoe, Dennis J,et al. | The amyloid hypothesis of Alzheimer's disease at 25years | EMBO MOLECULAR MEDICINE | 3028 | 2016 | 10.15252/emmm.201606210 |
| 3 | Heneka, Michael T,et al. | Neuroinflammation in Alzheimer's disease | LANCET NEUROLOGY | 2985 | 2015 | 10.1016/S1474-4422(15)70016-5 |
| 4 | Alvarez-Erviti, Lydia,et al. | Delivery of siRNA to the mouse brain by systemic injection of targeted exosomes | NATURE BIOTECHNOLOGY | 2783 | 2011 | 10.1038/nbt.1807 |
| 5 | Zlokovic, Berislav V | The blood-brain barrier in health and chronic neurodegenerative disorders | NEURON | 2199 | 2008 | 10.1016/j.neuron.2008.01.003 |
| 6 | Keren-Shaul, Hadas,et al. | A Unique Microglia Type Associated with Restricting Development of Alzheimer's Disease | CELL | 1939 | 2017 | 10.1016/j.cell.2017.05.018 |
| 7 | Guerreiro, Rita,et al. | TREM2 Variants in Alzheimer's Disease | NEW ENGLAND JOURNAL OF MEDICINE | 1790 | 2013 | 10.1056/NEJMoa1211851 |
| 8 | Halle, Annett,et al. | The NALP3 inflammasome is involved in the innate immune response to amyloid-beta | NATURE IMMUNOLOGY | 1678 | 2008 | 10.1038/ni.1636 |
| 9 | Heneka, Michael T,et al. | NLRP3 is activated in Alzheimer's disease and contributes to pathology in APP/PS1 mice | NATURE | 1564 | 2013 | 10.1038/nature11729 |
| 10 | Baker, Matt,et al. | Mutations in progranulin cause tau-negative frontotemporal dementia linked to chromosome 17 | NATURE | 1475 | 2006 | 10.1038/nature05016 |
| 11 | Hong, Soyon,et al. | Complement and microglia mediate early synapse loss in Alzheimer mouse models | SCIENCE | 1444 | 2016 | 10.1126/science.aad8373 |
| 12 | Yoshiyama, Yasumasa,et al. | Synapse loss and microglial activation precede tangles in a P301S tauopathy mouse model | NEURON | 1250 | 2007 | 10.1016/j.neuron.2007.01.010 |
| 13 | Cryan, John F,et al. | THE MICROBIOTA-GUT-BRAIN AXIS | PHYSIOLOGICAL REVIEWS | 1180 | 2019 | 10.1152/physrev.00018.2018 |
| 14 | Farris, W,et al. | Insulin-degrading enzyme regulates the levels of insulin, amyloid beta-protein, and the beta-amyloid precursor protein intracellular domain in vivo | PROCEEDINGS OF THE NATIONAL ACADEMY OF SCIENCES OF THE UNITED STATES OF AMERICA | 1142 | 2003 | 10.1073/pnas.0230450100 |
| 15 | Lim, GP,et al. | The curry spice curcumin reduces oxidative damage and amyloid pathology in an Alzheimer transgenic mouse | JOURNAL OF NEUROSCIENCE | 1139 | 2001 | 10.1523/JNEUROSCI.21-21-08370.2001 |
